# Risk factors of prolonged mechanical ventilation after acute type A aortic dissection surgery: a single-center retrospective study

**DOI:** 10.3389/fcvm.2025.1659336

**Published:** 2025-11-13

**Authors:** Guanying Chen, Zhenyu Li, Zhonglin Lin, Quanlin Su, Yun Ling, Tianbao Li, Chengbin Zhou

**Affiliations:** 1 Department of Cardiovascular Surgery, Guangdong Provincial Cardiovascular Institute, Guangdong Academy of Medical Sciences, Guangdong Provincial People's Hospital, South Medical University, Guangzhou, Guangdong, China; 2 Qiqihar Medical University, Qiqihar, Heilongjiang, China

**Keywords:** extubation, acute type A aortic dissection, risk factors, delay, mortality

## Abstract

**Objective:**

To identify the risk factors of prolonged mechanical ventilation after acute type A aortic dissection (ATAAD) surgery.

**Methods:**

466 patients undergoing ATAAD surgery from 2016.01 to 2021.01were studied retrospectively. Risk factors of delayed extubation (>48 h of ventilation) were identified using univariate and multivariate logistic regression. LASSO regression was used for variable selection. The ICU stay, hospital stay, complications, and 30-day mortality were analyzed.

**Results:**

Among the patients, 72% experienced delayed extubation. Risk factors included age (OR 1.042, 95% CI 1.005–1.083, *P* = 0.031), postoperative serum creatinine (Scr, OR 1.006, 95% CI 1.003–1.010, *P* < 0.001), and acute lung injury (ALI, OR 5.725, 95% CI 2.777–12.148, *P* < 0.001). Delayed extubation was associated with increased ICU stays, hospital stays, and complications, including MODS and higher mortality.

**Conclusions:**

Age, postoperative serum creatinine, and acute lung injury are significant risk factors for prolonged ventilation. Early identification and management of these factors may improve postoperative outcomes.

## Introduction

Acute type A aortic dissection (ATAAD) is a life-threatening and urgent condition affecting the large blood vessels, which can have severe consequences if the dissection leads to rupture (1). Aortic dissection happens when damage to the aorta's inner layer leads to blood flowing between the aortic wall layers, causing them to separate (2). Aortic dissection can be fatal because it causes insufficient blood flow and the possibility of aorta or heart rupture. Emergency surgery is required for Stanford type A dissection, which progresses quickly and has a poor prognosis. Due to the complexity of aortic dissection, even if surgery allows the patient to survive temporarily, the occurrence of some later complications can still pose a life-threatening risk to the patient, such as acute kidney injury, lung injury, and hypoxemia (3). Following cardiac surgery, mechanical ventilation is commonly applied as a postoperative measure to decrease both the myocardial oxygen consumption and the workload associated with spontaneous ventilation. Due to factors such as varying degrees of lung dysfunction (4, 5), gas exchange abnormalities, poor pulmonary mechanics and extracorporeal circulation (6), patients with ATAAD are prone to postoperative hypoxemia and pulmonary complications, which affects the duration of mechanical ventilation (7, 8). Prolonged ventilator support due to respiratory failure is a frequent and serious complication in patients undergoing ATAAD which also accounts for a significant number of in-hospital deaths (9). Therefore, identifying, preventing, and managing the duration of mechanical ventilation in ATAAD patients is crucial.

## Methods

### Study population

The consecutive patients who underwent ATAAD from January 2016 to December 2021 were studied retrospectively in single center. The patients meet the following criteria: TAAD suspected by aortic enhanced CT who underwent surgical treatment, onset of disease within 14 days, and age above 18 years. Patients who had traumatic aortic dissection, experienced aortic dissection during pregnancy, onset of acute type A aortic dissection (ATAAD) beyond 14 days prior to admission, experienced fatalities during the surgery or were under 18 years of age, were excluded from the study.

This study adhered to the ethical principles outlined in the Declaration of Helsinki and received approval from the Ethics Committee of Guangdong Provincial People's Hospital (KY2023- 187-01). Given the observational and retrospective nature of the study, the requirement for informed consent was waived by the Ethics Committee of Guangdong Provincial People's Hospital (KY2023- 187-01).

Baseline data were collected, which included demographics, comorbidities, preoperative laboratory values, and surgical procedure documentation. The demographic information included age, gender, body mass index (BMI), history of smoking and drinking; comorbidities including atria fibrillation, hypertension, hyperlipidemia, diabetes mellitus, chronic kidney disease, COPD; laboratory data contained RBC, WBC, PLT, Ddimer, glucose, AST, ALT, LDL, Tirglyceride and Scr.

### Data definition

Delayed extubation was defined as the total duration of mechanical ventilation exceeding 48 h after surgery. However, there is no universally accepted definition for delayed extubation after ATAAD surgery (7, 10). Hence, we adopted this particular definition based on previous studies and our institutional expertise. The secondary endpoints encompassed duration of ICU and hospital stays, various complications and mortality. Early mortality was categorized as deaths that occurred within 30 days following the hospitalization period subsequent to a surgical procedure. Stroke was charaterized as a prolonged central neurological deficit lasting beyond 72 h. The term “acute hepatic injury” was characterized by a notable increase in alanine aminotransferase levels surpassing tenfold the upper boundary of the standard range. Renal failure is defined as either the initiation of dialysis or a rise in serum creatinine levels to over 2.0 mg/dL, accompanied by a twofold increase compared to the most recent preoperative creatinine level. The definition of lung injury is that the chest imaging shows pulmonary infiltrates and the oxygenation index (PaO2/FiO2) is less than 300 mmHg.

### Surgical procedure

The procedures were performed using a median sternotomy approach and total cardiopulmonary bypass (CPB) with antegrade selective cerebral perfusion (SCP). Cannulation of the right axillary artery and femoral artery was utilized for CPB. During the cooling phase, the ascending aorta was clamped, followed by a longitudinal opening of the proximal ascending aorta. Antegrade perfusion of cold blood cardioplegic solution was directly infused into the coronary ostia during this period. Aortic root procedures were performed concurrently in this cooling phase. Circulatory arrest was initiated at a nasopharyngeal temperature of 20–25.8 degrees Celsius. Unilateral selective cerebral perfusion (SCP) was established via the right axillary artery following crossclamping of the brachiocephalic arteries. Crossclamping was also performed on the left carotid and left subclavian arteries. Rewarming started after completion of surgical operation.

### Statistical analysis

The statistical analysis in this study utilized SPSS software (version 26.0) and R software (version 4.0.3). Descriptive statistics were conducted to analyze both categorical and continuous variables. Categorical variables were reported as *n* (%) while continuous variables were reported as mean ± standard deviation or median (interquartile range). The Chisquare or Fisher exact test was used to compare categorical variables, the Mann–Whitney U test was used for non-normal variables, and the Student's T test was used for normally distributed variables.

We utilized clinical data to analyze the risk factors of delayed extubation in patients following ATAAD administration. Univariate analysis was performed to identify variables that could potentially be associated with delayed extubation in the cohort. We employed the least absolute shrinkage and selection operator (LASSO) method to identify the most informative predictive features from the primary dataset. This method is particularly useful for dimensionality reduction in high-dimensional data. By utilizing a LASSO binary logistic regression model, we were able to narrow down the pool of clinicopathologic variables in the cohort to a subset of potential predictors. When the penalization coefficient lambda (*λ*) is high, the estimated regression parameters remain unaffected; however, as the coefficients decrease, certain coefficients may shrink to zero. To determine the optimal *λ* in the LASSO model, we performed 10-fold cross-validation using minimum criteria and applied the one standard error of the minimum criteria (the 1-SE criterion). Variables were included in the multivariate logistic regression analysis.

## Results

### Baseline characteristics

**Figure 1:**
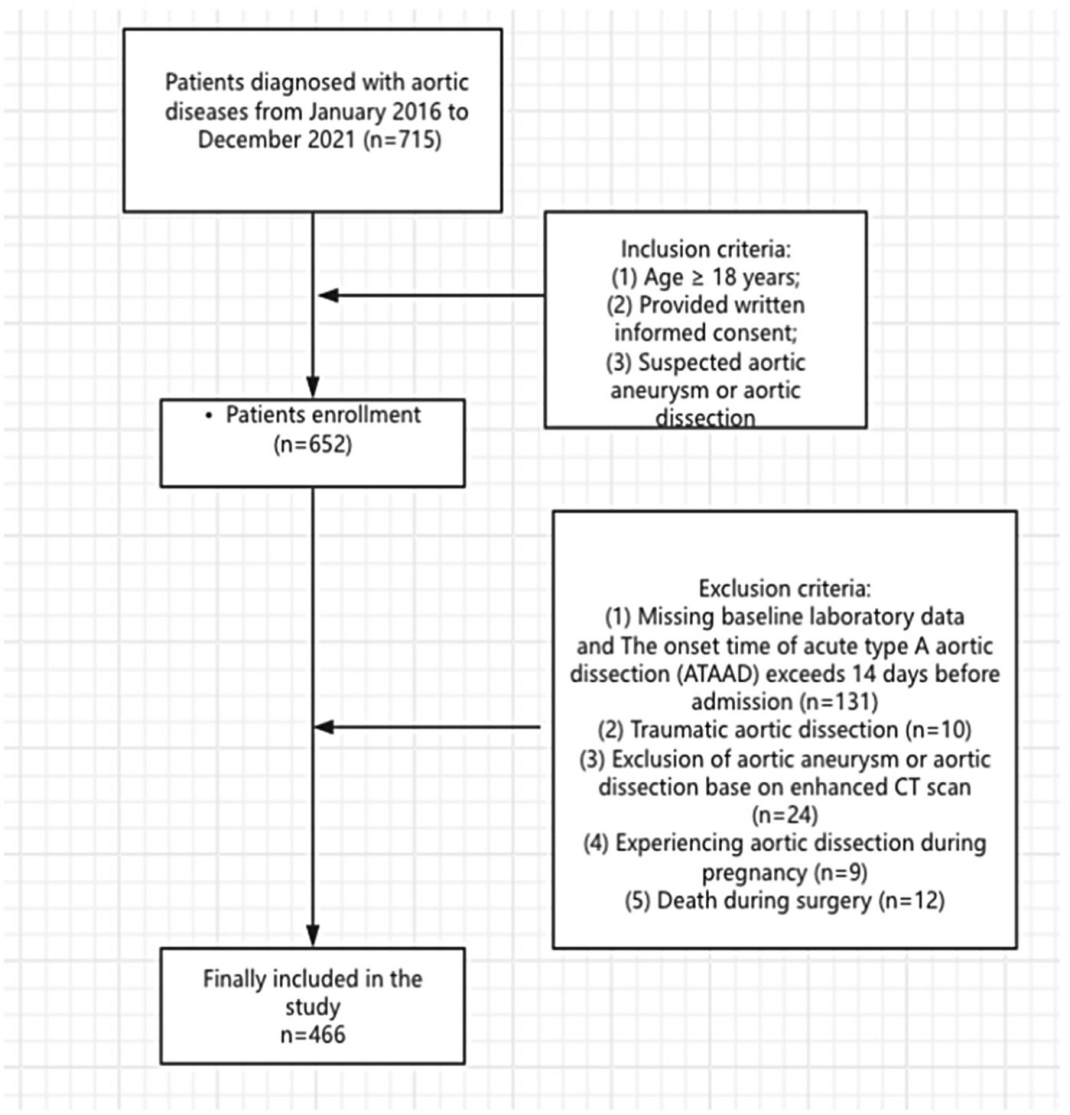
Flow diagram of patient inclusion and exclusion according to STROBE guidelines.

During a 5-year period in our institution, a total of 715 patients underwent type A acute aortic dissection repair surgeries. However, we excluded 249 patients, leaving us with 466 patients for the analysis (Figure 1). Out of the 466 patients included in the analysis, delayed extubation occurred in 336 patients (72%) after repair of acute type A aortic dissection. The average age of the entire cohort was 51.69 years, with males comprising 83.69% of the subjects. The average body mass index was 24.91 kg/m^2^, and 16.38% of the patients had a history of smoking. Furthermore, variables such as hypertension was observed in over 50% of the study population. Diabetes mellitus, chronic kidney disease, smoking and COPD were observed in less than 20% of the study population (Table 1).

**Table 1:** Preoperative data.

*n*	All patients(*n* = 466)	No delayed extubation (*n* = 130)	Delayed extubation (*n* = 336)	*p*
Age, mean(±SD)	51.689 ± 10.535	49.131 ± 10.723	52.679 ± 10.291	0.00
BMI, mean(±SD)	24.914 ± 3.846	24.383 ± 3.873	25.156 ± 3.809	0.15
Male, *n* (%)	390 (83.691)	109 (83.846)	281 (83.631)	0.96
Medical history				
Atria_fibrillation, *n* (%)	11 (2.439)	5 (3.937)	6 (1.852)	0.20
Hypertension, *n* (%)	303 (65.161)	76 (58.462)	227 (67.761)	0.06
Hyperlipidemia, *n* (%)	205 (44.469)	65 (50.388)	140 (42.169)	0.11
Diabetes mellitus, *n* (%)	17 (3.656)	2 (1.538)	15 (4.478)	0.13
Chronic kidney disease, *n* (%)	19 (4.086)	4 (3.077)	15 (4.478)	0.49
Smoking, *n* (%)	76 (16.379)	24 (18.462)	52 (15.569)	0.45
COPD, *n* (%)	53 (11.422)	18 (13.953)	35 (10.448)	0.29
Preoperative laboratory data				
RBC, mean (±SD)	143.557 ± 82.659	147.862 ± 81.793	141.887 ± 82.932	0.49
WBC, mean (±SD)	194.548 ± 118.016	190.392 ± 117.897	196.161 ± 118.022	0.64
PLT, mean (±SD)	150.183 ± 88.079	154.100 ± 82.157	148.663 ± 90.227	0.55
Ddimer, mean (±SD)	103.099 ± 82.115	115.069 ± 79.379	98.454 ± 82.686	0.05
Glucose, mean (±SD)	180.194 ± 104.778	175.354 ± 102.983	182.072 ± 105.406	0.54
AST, mean (±SD)	73.908 ± 37.630	73.077 ± 38.298	74.230 ± 37.362	0.77
ALT, mean (±SD)	63.170 ± 31.249	58.462 ± 31.006	64.997 ± 31.152	0.04
LDL, mean (±SD)	65.584 ± 22.335	65.760 ± 25.542	65.515 ± 20.948	0.92
Tirglyceride, mean (±SD)	57.148 ± 20.186	58.674 ± 22.086	56.552 ± 19.360	0.31
Scrpre, mean (±SD)	216.699 ± 126.065	213.569 ± 128.216	217.913 ± 125.199	0.74

RBC, red blood cell; WBC, white blood cell; ALT, alanine aminotransferase; AST, aspartate aminotransferase; PLT, platelets; Scr, serum creatinine; COPD, chronic obstructive pulmonary disease.

#### Intraoperative and postoperative conditions

Intraoperative conditions was showen in Table 2. Delayed extubation group had longer peration time, CPB time and cross clamp time. But no statistically significant differences were observed between the two groups in terms of other variables. Early Mortality, stroke, CRRT, reexploration for bleeding delayed chest closure, MODS, ECMO, acute lung injury, acute liver injury ccurred more frequently in the delayed extubation group. Patients with delayed extubation had a higher RBC count, WBC count, PLT count, Ddimer count and Scr postoperatively. Delayed extubation group had longer hospital stay and ICU stay day (Tables 3, 4).

**Table 2:** Intraoperative conditions.

*n*	All patients(*n* = 466)	No delayed extubation (*n* = 130)	Delayed extubation (*n* = 336)	*p*
Cannulation, *n* (%)				0.197
Axillary artery	281 (60.954)	77 (60.630)	204 (61.078)	
Femoral artery	24 (5.206)	9 (7.087)	15 (4.491)	
Axillary + femoral artery	132 (28.633)	31 (24.409)	101 (30.240)	
Others	24 (5.206)	10 (7.874)	14 (4.192)	
Bentall procedure without valve conduit, *n* (%)	23 (27.711)	9 (37.500)	14 (23.729)	0.20
Wheats, *n* (%)	9 (10.345)	2 (8.333)	7 (11.111)	0.70
Cabrol, *n* (%)	59 (56.731)	14 (45.161)	45 (61.644)	0.12
CABG, *n* (%)	22 (100.000)	4 (100.0 00)	18 (100.000)	1.000
Operation time, hour, mean (±SD)	7.500 ± 1.539	7.184 ± 1.511	7.621 ± 1.533	0.01
CPB time, min, mean (±SD)	245.467 ± 68.058	230.177 ± 73.962	251.400 ± 64.657	0.00
ACC time min, mean (±SD)	132.144 ± 42.620	124.354 ± 44.615	135.167 ± 41.427	0.01
DHCA tempnasal, mean (±SD)	23.222 ± 2.604	23.219 ± 2.854	23.223 ± 2.500	0.99
DHCAtime, mean (±SD)	39.487 ± 18.493	40.238 ± 17.626	39.195 ± 18.811	0.586
RBC transfusion, U, mean (±SD)	3.183 ± 3.529	2.995 ± 3.220	3.255 ± 3.639	0.52
Plasma transfusion, mL, mean (±SD)	232.468 ± 261.013	210.748 ± 249.956	240.827 ± 264.671	0.31
Platele transfusion, U, mean (±SD)	1.941 ± 3.108	1.680 ± 2.286	2.041 ± 3.366	0.31

CPB, cardiopulmonary bypass; ACC, aortic cross clamp; CABG, coronary artery bypass graft; DHCA, deep hypothermic circulatory arrest.

**Table 3:** Postoperative conditions.

*n*	All patients (*n* = 466)	No delayed extubation (*n* = 130)	Delayed extubation (*n* = 336)	*p*
Early Mortality, *n* (%)	39 (8.387)	5 (3.876)	34 (10.119)	0.03
Stroke, *n* (%)	48 (10.300)	7 (5.385)	41 (12.202)	0.03
CRRT, *n* (%)	120 (25.751)	6 (4.615)	114 (33.929)	<0.001
Reexploration for bleeding delayed chest closure, *n* (%)	46 (9.871)	5 (3.846)	41 (12.202)	0.01
MODS, *n* (%)	147 (31.613)	17 (13.178)	130 (38.690)	<0.001
ECMO, *n* (%)	22 (4.721)	2 (1.538)	20 (5.952)	0.04
Acute lung injury, *n* (%)	325 (69.742)	53 (40.769)	272 (80.952)	<0.00
Acute Liver Injury, *n* (%)	208 (44.635)	45 (34.615)	163 (48.512)	0.01
Postoperative laboratory data				
RBC, median [IQR]	3.010 [2.600,3.550]	3.180 [2.840,3.670]	2.950 [2.510,3.450]	<0.001
WBC, median [IQR]	20.090 [16.710,24.060]	19.140 [16.400,22.860]	20.320 [17.190,24.830]	0.019
PLT, median [IQR]	106.000 [65.000,143.000]	131.000 [98.000,178.000]	98.000 [56.000,129.000]	<0.001
Ddimer, mean (±SD)	9,891.473 ± 6,311.687	7,419.587 ± 5,647.908	10,870.899 ± 6,293.054	<0.001
Glucose, median [IQR]	12.530 [10.010,15.490]	11.470 [9.780,15.420]	12.730 [10.270,15.520]	0.118
AST, mean (±SD)	401.393 ± 1,615.408	234.496 ± 1,091.381	464.231 ± 1,768.942	0.176
ALT, mean (±SD)	271.063 ± 751.591	184.124 ± 599.790	304.641 ± 800.045	0.122
Scr, median [IQR]	186.920 [123.350,350.860]	125.800 [93.000,173.200]	240.200 [142.300,424.660]	<0.001
ICU stay day, median [IQR]	9.000 [6.000,20.000]	5.000 [3.000,10.000]	11.000 [8.000,24.000]	<0.001
Hospital stay, day, median [IQR]	21.000 [15.000,29.000]	18.000 [14.000,25.000]	22.000 [16.000,30.000]	<0.001

RBC, red blood cell; WBC, white blood cell; ALT, alanine aminotransferase; AST, aspartate aminotransferase; PLT, platelets; Scr, serum creatinine; COPD, chronic obstructive pulmonary disease; MODS, multiple organ dysfunction syndromes; CRRT, continuous renal replacement therapy; ICU, intensive care unit.

**Table 4:** Univariable analysis of the risk factors for delayed extubation.

Predictor	OR	95%CI	*P*-value
Age	1.033	[1.013,1.053]	0.001
BMI	1.055	[0.982,1.133]	0.145
Male	0.784	[0.389,1.489]	0.473
Preoperative laboratory data			
RBC	0.999	[0.997,1.002]	0.484
WBC	1.000	[0.999,1.002]	0.636
PLT	0.999	[0.997,1.002]	0.550
D-dimer	0.998	[0.995,1.000]	0.051
Glucose	1.001	[0.999,1.003]	0.535
AST	1.001	[0.995,1.006]	0.767
ALT	1.007	[1.000,1.013]	0.044
LDL	1.000	[0.990,1.009]	0.916
Tirglyceride	0.995	[0.985,1.005]	0.312
Scr	1.000	[0.999,1.002]	0.739
CPB time	1.006	[1.002,1.009]	0.003
ACC time	1.006	[1.001,1.012]	0.015
DHCA time	0.997	[0.986,1.008]	0.585
DHCA tempnasal	1.001	[0.926,1.081]	0.990
RBC transfusion, U	1.021	[0.958,1.089]	0.517
Plasma transfusion, mL	1.000	[1.000,1.001]	0.312
Platelets transfusion, U	1.044	[0.960,1.136]	0.313
Postoperative laboratory data			
RBC	0.998	[0.992,1.004]	0.520
WBC	1.053	[1.017,1.091]	0.003
PLT	0.991	[0.988,0.995]	<0.001
Ddimer	1.000	[1.000,1.000]	<0.001
Glucose	1.041	[0.991,1.094]	0.109
AST	1.000	[1.000,1.000]	0.213
ALT	1.000	[1.000,1.001]	0.141
Scr	1.006	[1.004,1.009]	<0.001
Blood loss, mL	1.001	[1.000,1.003]	0.021
Medical history			
Atria_fibrillation	0.460	[0.138,1.536]	0.207
Hypertension	1.493	[0.984,2.267]	0.060
Diabetes mellitus	3.000	[0.676,13.305]	0.148
Hyperlipidemia	0.718	[0.477,1.080]	0.112
Chronic kidney disease	1.477	[0.481,4.535]	0.496
Smoking	0.814	[0.478,1.387]	0.450
COPD	0.719	[0.391,1.322]	0.289
Cannulation			
Femoral vs. axillary	0.629	[0.264,1.497]	0.295
Femoral + axillary vs. axillary	1.230	[0.761,1.988]	0.399
Othera vs. axillary	0.528	[0.225,1.240]	0.143
Postoperative outcomes			
Stroke	3.203	[1.226,8.372]	0.018
CRRT	9.064	[3.838,21.407]	<0.001
Re-exploration for bleeding delayed chest closure	3.106	[1.187,8.129]	0.021
MODS	4.161	[2.354,7.357]	<0.001
ECMO	3.394	[0.771,14.945]	0.106
Mediastinitis	0.630	[0.148,2.684]	0.532
Acute lung injury	6.055	[3.728,9.833]	<0.001
Acute liver injury	1.744	[1.103,2.757]	0.017

#### Feature selection

**Figure 2:**
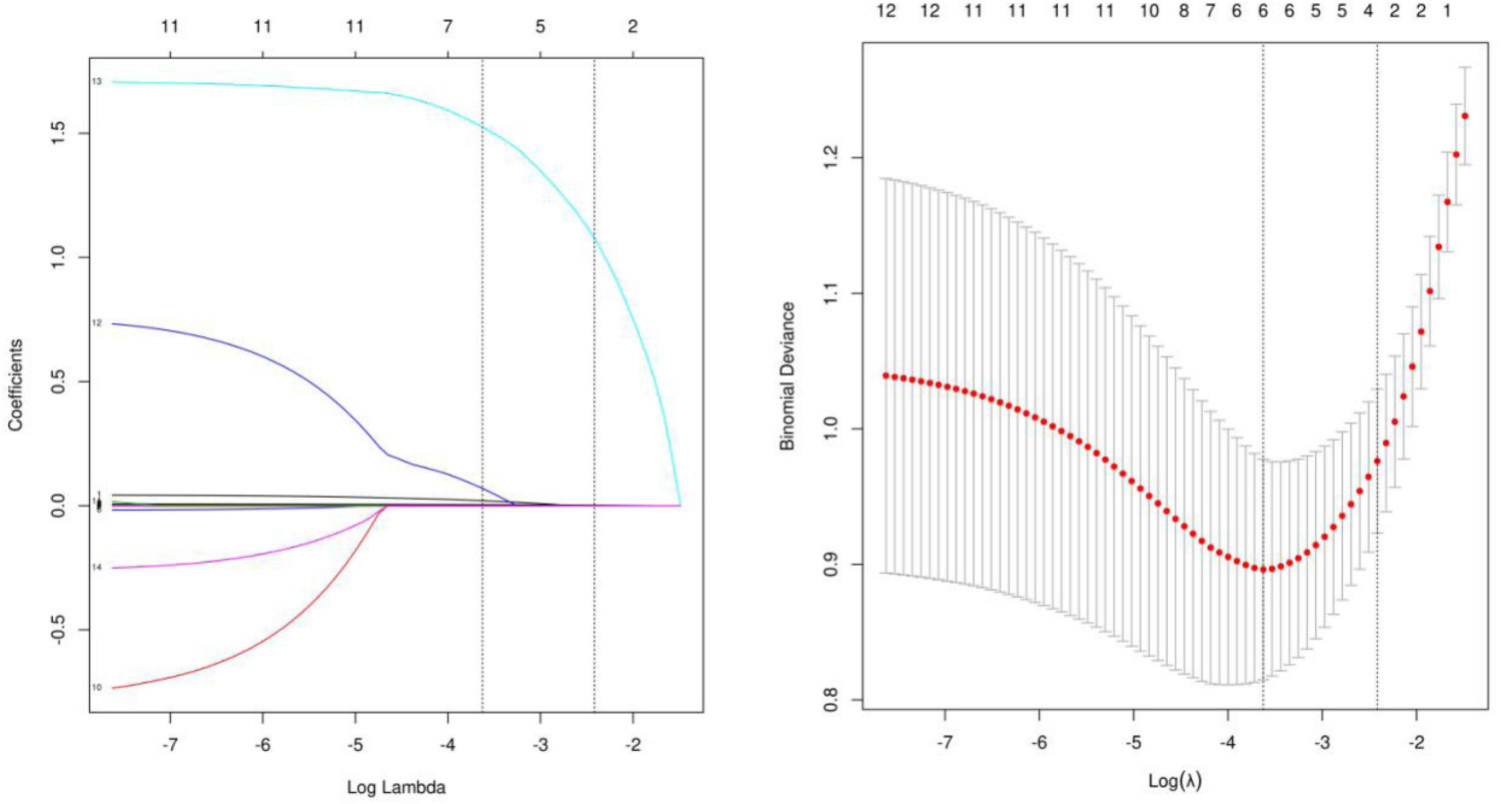
Demographic and clinical feature selection using lasso binary logistic regression model. **(A)** Tuning parameters *λ* were obtained through 10 times crossvalidation with the minimum standard, the partial likelihood deviance curve was drawn through Log (*λ*), and the minimum standard and 1-SE standard were used to draw the vertical dotted line, after 10 times cross-validation, the Log (*λ*) was determined to be 0.027 (1-SE standard) at the optimal value. **(B)** Lasso coefficient profiles of the 9 features. A coefficient profile plot was produced against the log (*λ*) sequence. The vertical line was drawn at the value selected using 10fold cross-validation, where optimal resulted in 6 features with non-zero coefficients.

The LASSO binary logistic regression model was utilized due to the insufficient sample size in this study, which did not meet the recommended number of events per variable. Out of all the relevant variables, a cohort-based analysis resulted in the reduction of 9 features to 6 potential predictors. The final model utilized the 6 variables with non-zero coefficients in the LASSO logistic regression model, namely Age, PLTpost, postoperative D-dimer level, MODS, Acute lung injury and Scrpost (Figures 2A,B). Subsequently, the aforementioned 6 variables were incorporated into a multivariable logistic regression analysis to identify the risk factors associated with delayed extubation. The analysis revealed that age, acute lung injury and Scrpost were significant risk factors for delayed extubation (Table 5).

**Table 5:** Univariable analysis of the risk factors for delayed extubation.

Predictor	Odds Ratio	Lower	Upper	*p*
(Intercept)	0.048	0.005	0.427	0.008
Age	1.042	1.005	1.083	0.031
PLTpost	0.995	0.99	1.0	0.055
Scrpost	1.006	1.003	1.01	<0.001
Acute lung injury	5.725	2.777	12.148	<0.001

#### Nomogram construction

**Figure 3:**
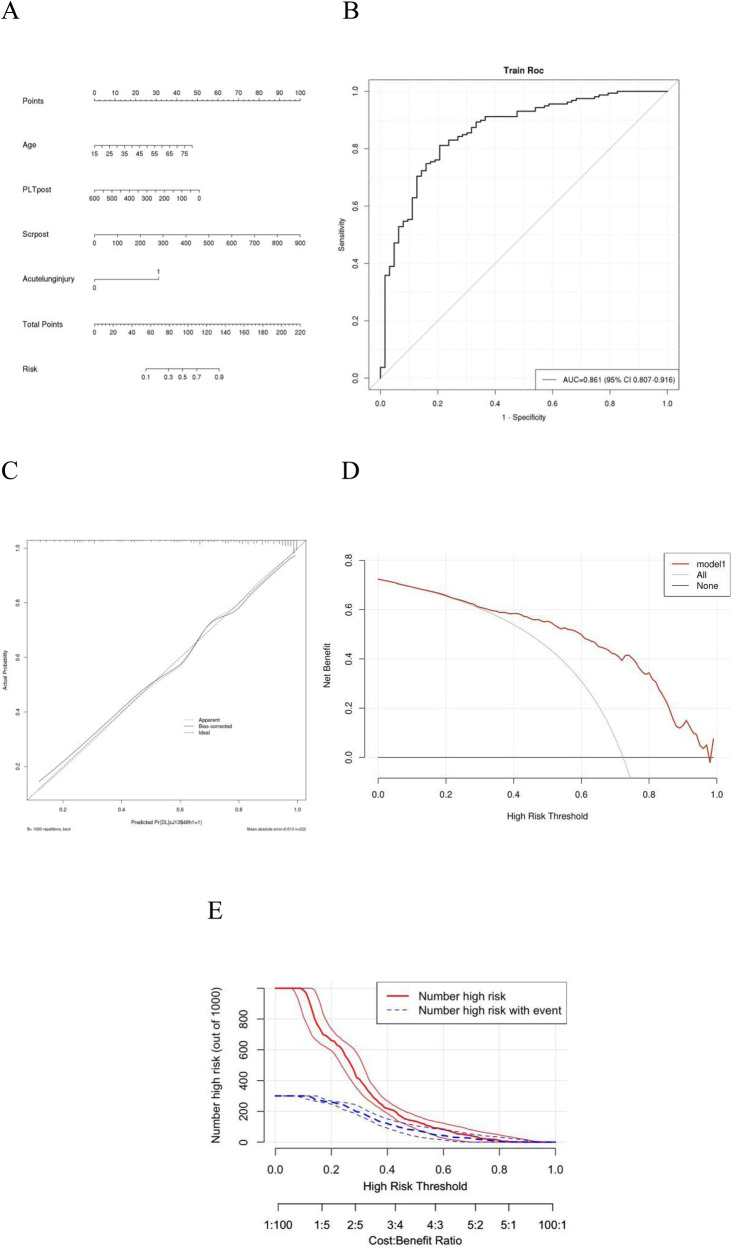
ROC analysis, calibration curves and DCA for delayed extubation. Plots **(A)** shows the nomogram analysis of delayed extubation. Plots **(B)** shows the ROC analysis of delayed extubation. Plots **(C)** Calibration curves of the prediction model. Calibration curves depicted the calibration of the model. The 45◦ line represents a perfect prediction, and the red line and green line represent the actual prediction and bootstrap adjusted curve respectively. Plots **(D)** show DCA of Model for delayed extubation. Plots **(E)** show clinical impact curve of Model for delayed extubation. AUC, area under the receiver operating characteristic curve; CI, confidence interval; DCA, decision curve analysis; ROC, receiver operating characteristic.

A backwards selection logistic regression was performed to screen the aforementioned variables. And AUC was 0.86 (Figure 3B). Age, acute lung injury and Scrpost were identified as risk factors for delayed extubation (Table 5). These findings were utilized to develop a nomogram that can estimate the risk of delayed extubation in patients with aortic dissection during their hospitalization (Figures 3A,B). Figure 3C displayed the calibration curves of the nomogram. In the cohort, the calibration curves demonstrated nearly diagonal alignment, indicating that the nomogram had a good fit. The decision curve analysis revealed that our predictive model yielded greater clinical net benefits when compared to both the “intervention for all” and “no intervention” strategies (Figure 3D). The clinical impact curve also demonstrated that the model exhibited favorable predictive capacity and clinical usefulness (Figure 3E).

## Discussion

This study revealed a prevalence higher rate of delayed extubation in patients undergoing AADS, which was not similar to data that had been previously reported (9, 11, 12). We observed higher resource utilization and in-hospital mortality among patients with delayed extubation after AADS, as compared to those without delayed extubation. The in-hospital mortality rate for patients undergoing delayed extubation a AADS was 10.12%, which was less than the findings from previous studies on cardiac surgical patients (9, 13).

Our study examined the predictors of delayed extubation in patients with AADS and develop a nomogram model for predicting the complication. Based on clinical data from 466 patients who underwent AADS at a single institution, it was found that certain preoperative factors such as age, along with postoperative factors including age, SCr (Serum creatinine), and acute lung injury, were independently associated with delayed extubation following AADS. Our predictive model was developed based on these risk factors and subsequently validated, demonstrating excellent predictive performance and strong clinical utility. And the ACU was 0.86 therefore the prediction is relatively good.

It was observed that older age was associated with a higher risk of delayed extubation in our study population. However, the findings regarding the relationship between age and the probability of tracheostomy varied across different studies (9, 14, 15). We believe that this inconsistency could be attributed, at least in part, to variations in the disease populations under investigation. And we also found that there was no significant correlation between postoperative platelet count and delayed extubation, with a *p*-value of 0.055. Increasing the sample size may lead to different conclusions. Similarly, during univariate analysis, no correlation was found between preoperative platelet levels and delayed extubation. However, some other studies have found a correlation between preoperative platelet levels and delayed extubation (10). And the other found the transfusion of blood products had a significant correlation with the duration of delayed extubation. The incidence of delayed extubation was found to increase with the transfusion of packed red blood cells (pRBC) and platelet concentrate (PC), as well as with advancing age, across all three endpoints (15).

In our study, we found that postoperative creatinine has a good predictive role in delayed extubation, which was not discovered in previous research. However, increased serum cystatin C levels upon admission were identified as risk factors for delayed extubation in one research study (16). The researcher identified elevated leukocyte count, lower preoperative platelet count, and longer cardiopulmonary bypass (CPB) duration as risk factors for delayed extubation. However, this study only conducted univariate logistic regression without incorporating multivariate logistic regression (16). The impact of creatinine on aortic dissection has been extensively investigated in previous research. Earlier studies primarily examined creatinine as a predictive factor for postoperative renal injury (17), while more recent studies have discovered its potential as a reliable predictor of mortality (18–21). Creatinine, primarily eliminated via glomerular filtration, represents the metabolic product of muscle-produced creatine. Serum creatinine (SCr) remains unaffected by exogenous factors including diet and intense catabolism, as it is under homeostatic regulation through glomerular filtration (21). The association between SCr and delayed extubation was uncovered by our study, and further investigation is required to understand its underlying mechanism. The possible reasons are that decreased renal function leads to fluid retention and positive fluid balance, which increases pulmonary capillary hydrostatic pressure and results in interstitial and alveolar edema, thereby reducing gas exchange and prolonging the time to extubation (22). Additionally, renal dysfunction causes an imbalance in the immune response (with both immunosuppression and inflammation present), increasing the risk of hospital-acquired or pulmonary infections, and thus prolonging mechanical ventilation (23).

The study has some limitations, including a single centre study, relatively small dataset and a lack of long-term follow-up. We will continue with further investigation in a subsequent study. Significant differences in operation time, CPB time, and ACC time were noted between groups. These parameters are biologically relevant to PMV. It's possible that I didn't include these variables in my Lasso regression analysis. Perhaps with an expanded sample size in the future, these factors might be incorporated as well. I will address this in the limitations section.

In conclusion, Age, postoperative serum creatinine, and acute lung injury are significant risk factors for prolonged ventilation. Early identification and management of these factors may improve postoperative outcomes.

## Data Availability

The raw data supporting the conclusions of this article will be made available by the authors, without undue reservation.
