# Correction: Risk factors of prolonged mechanical ventilation after acute type A aortic dissection surgery: a single-center retrospective study

**DOI:** 10.3389/fcvm.2026.1794436

**Published:** 2026-02-23

**Authors:** Guanying Chen, Zhenyu Li, Zhonglin Lin, Quanlin Su, Yun Ling, Tianbao Li, Chengbin Zhou

**Keywords:** extubation, acute type A aortic dissection, risk factors, delay, mortality

There was a mistake in Figure 1 as published. Figure 1 does not provide a detailed description of the inclusion and exclusion criteria. The corrected Figure 1 appears below.

**Figure 1:**
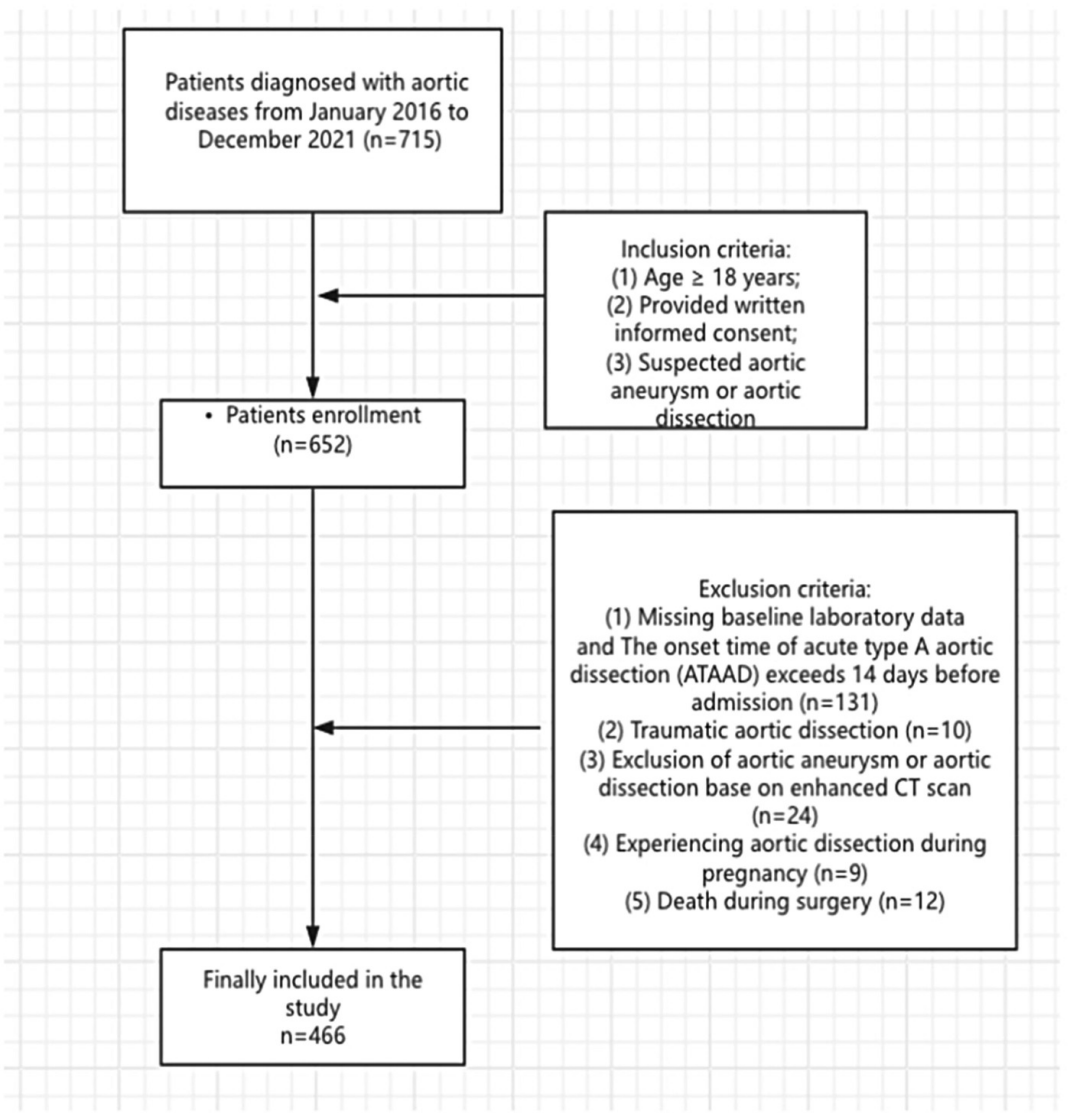
Flow diagram of patient inclusion and exclusion according to STROBE guidelines.

An incorrect Funding statement was provided. The correct funder is the Guangdong Precision Medicine Application Association.

The *Study population* section in the **Methods** is not detailed enough and needs to be revised. A correction has been made to the section **Methods,** *Study population,* Paragraph 1*:*

“The consecutive patients who underwent ATAAD from January 2016 to December 2021 were studied retrospectively in single center. The patients meet the following criteria: TAAD suspected by aortic enhanced CT who underwent surgical treatment, onset of disease within 14 days, and age above 18 years. Patients who had traumatic aortic dissection, experienced aortic dissection during pregnancy, onset of acute type A aortic dissection (ATAAD) beyond 14 days prior to admission, experienced fatalities during the surgery or were under 18 years of age, were excluded from the study.”

The last sentence in the *Data definition* section in the **Methods** is unclear regarding the definition of lung injury. A correction has been made to the section **Methods,** *Data definition:*

“Delayed extubation was defined as the total duration of mechanical ventilation exceeding 48 h after surgery. However, there is no universally accepted definition for delayed extubation after ATAAD surgery (7, 10). Hence, we adopted this particular definition based on previous studies and our institutional expertise. The secondary endpoints encompassed duration of ICU and hospital stays, various complications and mortality. Early mortality was categorized as deaths that occurred within 30 days following the hospitalization period subsequent to a surgical procedure. Stroke was charaterized as a prolonged central neurological deficit lasting beyond 72 h. The term “acute hepatic injury” was characterized by a notable increase in alanine aminotransferase levels surpassing tenfold the upper boundary of the standard range. Renal failure is defined as either the initiation of dialysis or a rise in serum creatinine levels to over 2.0 mg/dL, accompanied by a twofold increase compared to the most recent preoperative creatinine level. The definition of lung injury is that the chest imaging shows pulmonary infiltrates and the oxygenation index (PaO_2_/FiO_2_) is less than 300 mmHg.”

The original version of this article has been updated.

